# The impact of power on implicit attitudes toward violence: empathy as a mediator and moderator

**DOI:** 10.1186/s40359-026-04649-5

**Published:** 2026-05-01

**Authors:** Lin Guo, Xiang Yun

**Affiliations:** 1 Office of Academic Discipline Development, Jiangsu Second Normal University, Nanjing, 211200 China; 2 Department of Management, Nanjing Police University, Nanjing, 210023 China

**Keywords:** Power, State empathy, Trait empathy, Implicit attitude toward violence

## Abstract

**Background:**

Attitudes toward violence can be assessed using both explicit self-report measures and implicit paradigms. However, little is known about how situational power influences such implicit evaluations and how empathy shapes this process. Power has been identified as a potential factor influencing implicit attitudes toward violence, while empathy may serve as a protective buffer. This study aimed to examine how different forms of power affect implicit attitudes toward violence and to clarify the mediating and moderating roles of empathy.

**Methods:**

Two experimental studies were conducted. In Study 1, explicit power was primed through a recall task. Participants’ state empathy and implicit attitudes toward violence were subsequently measured. In Study 2, implicit power was primed using a conceptual word-search task, and trait empathy was assessed as a potential moderator. Both studies utilized the Implicit Association Test (IAT) to measure implicit violent attitudes.

**Results:**

In Study 1, participants primed with a high sense of power exhibited significantly lower state empathy, which was associated with less negative implicit evaluations of violence. In Study 2, trait empathy moderated the relationship between power and implicit evaluations of violence: lower levels of trait empathy were associated with less negative implicit evaluations of violence under power priming.

**Conclusion:**

These findings suggest that both explicit and implicit power priming are associated with shifts in implicitly measured evaluations of violence, and that empathy plays a key regulatory role. Specifically, state empathy showed a significant indirect effect in Experiment 1, whereas trait empathy functioned as a boundary condition in Experiment 2. These findings point to different roles of empathy in the association between power priming and implicitly measured evaluations of violence, and suggest potential implications for understanding how power relates to attitudes toward violence.

## Introduction

Attitudes toward violence encompass individuals’ cognitive evaluations and emotional dispositions regarding violent behavior. These attitudes serve as critical psychological predictors of actual aggression and violence [1, 2]. Understanding the antecedents of violent attitudes is essential for elucidating the psychological mechanisms that drive aggression, improving behavioral prediction, and informing effective intervention strategies. Traditionally, research in this area has relied on self-reported measures of explicit attitudes. However, such measures are often compromised by social desirability bias, as individuals may suppress or distort their true beliefs when addressing sensitive issues like violence [3]. To address this, scholars have increasingly tended to focus on implicitly measured evaluative tendencies, often termed “implicit attitudes.” In the present study, we conceptualize these attitudes through a functional-procedural lens [4, 5]. Rather than assuming an ontologically distinct or hidden mental system, we define the target construct as the automatically activated evaluations triggered by indirect measurement procedures (e.g., the IAT). As noted by Tahamata and Tseng [6], this approach emphasizes the procedural nature of the measurement rather than a fixed internal entity. Empirical evidence suggests that such automatic evaluations are reliable predictors of norm-sensitive behaviors [7], often demonstrating greater predictive validity than explicit measures in sensitive contexts [3, 8]. Building upon these considerations, the present study seeks to investigate how power influences these implicitly measured evaluations of violence and the underlying psychological mechanisms.

Empirical research has consistently shown that structural imbalances of power within interpersonal relationships—such as disparities in economic resources or social status—are strongly associated with violent behavior [9]. In intimate relationships, power asymmetry is a known risk factor: individuals with greater power are more likely to perpetrate violence [10–12], while those who perceive themselves as powerless are at increased risk of victimization and abuse [13, 14]. Similar dynamics have been observed in organizational settings, where power differentials often predict aggressive behavior from superiors toward subordinates [15] .

At the same time, it is important to acknowledge that power and the subjective sense of power are not inherently detrimental. A growing body of research suggests that experiencing personal power can have psychologically beneficial consequences, including increased subjective well-being, perceived control, and motivation toward valued goals [16–18]. Moreover, some studies indicate that power may under certain conditions promote prosocial orientation, empathic accuracy, and interpersonal sensitivity [19, 20]. These findings suggest that the psychological consequences of power are not uniform, but rather depend on how power is construed and the psychological processes it activates.

To better isolate these processes, researchers have increasingly focused on the subjective experience of power—referred to as the “sense of power.” Unlike structural or institutional power, the sense of power pertains to individuals’ perceived capacity to influence others [21] and can be experimentally manipulated through techniques such as autobiographical recall [21, 22]. Accumulating evidence indicates that variations in the sense of power systematically shape social cognition. Under certain conditions, heightened power has been associated with reduced attention to others’ perspectives [23], diminished emotional responsiveness [24], weakened adherence to social norms [18, 25], and increased objectification of others [26]. These shifts in social cognition may, in turn, create psychological conditions under which aggressive tendencies become more likely to emerge.

To examine how situational experiences of power influence psychological processes under controlled conditions, researchers commonly employ power priming paradigms to temporarily activate individuals’ subjective sense of power [21, 22]. Consistent with this perspective, previous research has shown that the sense of power is cognitively linked to violent concepts [27, 28], suggesting that power may increase the accessibility of aggression-related representations. However, cognitive accessibility alone does not necessarily imply a change in evaluative responses toward violence. Importantly, experimental studies directly examining evaluative outcomes suggest that temporarily activating a sense of power can weaken individuals’ negative implicit evaluations of violence [29] and increase the likelihood of retaliatory [30] and sexually aggressive behavior [31]. Taken together, these findings suggest that although power is not inherently maladaptive, certain experiences of power—particularly when they reduce interpersonal sensitivity and concern for others—may increase the risk of endorsing violence.

While the sense of power may serve as a psychological antecedent for violent behavior, empathy is widely recognized as a protective factor. Empathy is defined as the capacity to understand and share another person’s thoughts and emotional states from their perspective [32]. It plays a crucial role in inhibiting aggression and promoting prosocial behavior. Empirical studies consistently demonstrate a robust negative association between empathy and violence [33–36]. Deficits in empathy have been linked to various forms of antisocial and criminal conduct, including aggression, callous-unemotional traits, and delinquency [36, 37]. Recent work further suggests that reduced empathy is associated with systematic biases in social-cognitive and evaluative processing, including less charitable interpretations of others’ motives [38].

Empathy is not a unitary construct but can be conceptualized at different levels of psychological functioning, ranging from situationally fluctuating empathic states to relatively stable dispositional tendencies [39]. At the situational level, individuals may experience temporary variations in empathic concern depending on contextual cues and social experiences. At the dispositional level, empathy reflects relatively enduring personality characteristics that shape how individuals respond to others across situations. Recognizing these different levels of empathy is important for understanding how social experiences—such as feeling powerful—may influence social cognition and evaluative processes.

Building on this distinction, the present research examines how the sense of power may influence implicit evaluations of violence through both situational and dispositional pathways. Study 1 focuses on state empathy as a situational psychological mechanism linking power priming to implicit evaluations of violence. Existing research suggests that experiential inductions of power tend to reduce empathic concern and impair perspective-taking [21, 24]. Accordingly, we hypothesize that activating an explicit sense of power will reduce individuals’ momentary empathic responding, which in turn may weaken their negative implicit attitudes toward violence.

Study 2 extends this investigation by shifting the focus from situational processes to stable individual differences. Trait empathy represents a dispositional tendency to experience concern for others and has been widely identified as a protective factor against aggression and violence. Accordingly, Study 2 examines whether trait empathy moderates the relationship between power priming and implicit attitudes toward violence. Specifically, we hypothesize that individuals with higher levels of trait empathy will be less susceptible to the influence of power priming.

Taken together, the present research adopts a two-study design to examine empathy from complementary perspectives. Study 1 investigates whether state empathy functions as a situational mechanism linking power priming to implicit evaluations of violence, whereas Study 2 tests whether trait empathy serves as a dispositional boundary condition. By integrating these process-oriented and person-centered perspectives, the two studies provide a multi-level examination of empathy’s role in the relationship between power and implicit evaluations of violence.

## Experiment 1

The primary aim of Experiment 1 was to examine whether an explicitly activated sense of power influences individuals’ implicit attitudes toward violence through its impact on momentary empathic responding. Building on prior evidence that experiential power inductions can reduce perspective-taking and empathic concern [21, 24], this study employed an autobiographical recall task to manipulate participants’ subjective sense of power. We conceptualized empathy as a state-level psychological process and tested the hypothesis that power priming would decrease state empathy, which in turn would mediate the effect of power on implicit attitudes toward violence.

### Method

#### Materials and instruments

##### Power priming

The study employed a situational recall task to induce participants’ subjective sense of power [21]. Participants assigned to the high-power condition were instructed to write a brief essay (approximately 200 words) describing a situation in which they had control or influence over others. In contrast, those in the low-power condition were asked to recall a situation in which they were controlled by or dependent on others. This recall task served as the experimental manipulation of power in Experiment 1 and is widely used to induce a temporary subjective sense of power [21, 22].

##### Manipulation check for power priming

To assess the effectiveness of the power manipulation, four items adapted from Lammers et al. [40] were used to measure participants’ momentary sense of power (e.g., “Right now, I feel powerful,” “Right now, I feel influential”). Responses were recorded on a 7-point Likert scale. In the current sample, internal consistency for this measure was acceptable (α = 0.78).

##### State empathy

State empathy was assessed using four items adapted from Batson et al. [41], which measured participants’ current emotional sensitivity to others (e.g., “Right now, I feel sensitive to others’ emotions”). Responses were rated on a 7-point Likert scale. The internal consistency of this scale was α = 0.73.

##### Implicit attitudes toward violence

Implicit attitudes were measured using a Chinese version of the Violence Implicit Association Test (IAT), developed with Inquisit 3.0 by Li et al. [29]. The violence IAT paradigm was originally proposed by Gray et al. [42] and Snowden et al. [43], and has since been widely adopted in studies of aggression-related cognition. The target categories included “violent” and “peaceful” behaviors, while the attribute categories consisted of positive and negative evaluative words. Examples of target stimuli included words such as assault, robbery (violent) and chatting, walking (peaceful), and examples of attribute words included smile, comfort (positive) and fear, horror (negative). The IAT followed the standard 7-block structure outlined by Greenwald et al. [44], using the improved scoring and procedural recommendations proposed by Greenwald et al. [44] and Lane et al. [45]. The experimental structure and stimulus construction were consistent with previous violence IAT studies [29, 42, 43]. Higher D-scores indicated stronger negative implicit evaluations of violence. Higher D-scores indicated stronger negative implicit evaluations of violence. Given the structure of the IAT, a lower D-score reflects a smaller difference in reaction times between compatible and incompatible task pairings. Accordingly, lower IAT effects indicate weaker implicit associations between violence and negative valence. D-scores were calculated using a standard SPSS script available from the University of Washington IAT website.

#### Procedure

Participants first completed the power priming task, followed by the manipulation check assessing their momentary sense of power and the state empathy scale. Subsequently, they completed the Implicit Association Test (IAT) on a computer.

#### Participants

A total of 155 undergraduate students participated in the study. Prior to analysis, participants’ responses to the power recall task were screened based on predetermined exclusion criteria. Responses were considered invalid if they (a) did not describe a situation involving interpersonal influence or dependence as required by the task instructions, or (b) contained fewer than 100 Chinese characters, which was used as a minimal threshold to indicate insufficient engagement with the task. Based on these criteria, three participants were excluded due to invalid recall task responses. In addition, six participants whose error rates on the Implicit Association Test (IAT) exceeded 20%, consistent with established IAT data-cleaning recommendations [44], were excluded. The final sample comprised 146 valid participants (78 females), with a mean age of 21.2 years (SD = 1.85). Participants were randomly assigned to either the high-power priming condition (*n* = 74, 36 females) or the low-power priming condition (*n* = 72, 42 females).

### Results

#### Group differences in state empathy and implicit violence attitudes

For state empathy, an independent samples t-test revealed that participants in the low-power priming condition reported significantly higher scores (M = 5.33, SD = 0.79) than those in the high-power condition (M = 4.14, SD = 0.85), t(144) = 8.75, *p* < .001, d = 1.45. This effect of power condition on state empathy remained significant when age and gender were included as covariates in an ANCOVA, F(1,142) = 74.18, *p* < .001, η_p_² = 0.34, indicating that the power manipulation elicited distinct levels of empathic response.

Regarding implicit attitudes toward violence, a one-sample t-test indicated that the overall IAT D-scores were significantly greater than zero, t(145) = 53.74, *p* < .001, demonstrating a robust implicit association between violence and negative valence at the group level. Consistent with this, the vast majority of participants exhibited IAT effects in the expected direction. Moreover, the low-power group exhibited a higher IAT D-score (M = 1.25, SD = 0.21) than the high-power group (M = 0.99, SD = 0.22). This difference was also statistically significant, t(144) = 7.32, *p* < .001, d = 1.21. Critically, the group difference remained significant when controlling for age and gender in an ANCOVA, F(1, 142) = 51.95, *p* < .001, η_p_² = 0.27, suggesting that priming a sense of power influenced participants’ implicit evaluations of violence. Therefore, the reduced D-scores in the high-power condition suggest that participants held less negative implicit attitudes toward violence, implying that heightened power reduced cognitive conflict when associating violent concepts with positive attributes.

Additional analyses revealed no significant Power × Gender interactions on implicit attitudes, state empathy, or manipulation check (all ps > 0.10), indicating that the effects were comparable across male and female participants.

#### Mediating role of state empathy

To examine whether state empathy mediated the relationship between power priming and implicit attitudes toward violence, we conducted a mediation analysis using PROCESS 4.0 for SPSS (Model 4, 5,000 bootstrap samples), controlling for age and gender.

First, power priming significantly predicted state empathy, B = − 1.18, SE = 0.14, t(143) = − 8.61, *p* < .001, indicating that participants in the high-power condition reported lower levels of state empathy. State empathy, in turn, significantly predicted implicit evaluations of violence when controlling for power, B = 0.08, SE = 0.02, t(142) = 3.85, *p* < .001. Power priming also remained a significant predictor of implicit evaluations of violence when controlling for empathy, B = − 0.16, SE = 0.04, t(141) = − 3.89, *p* < .001, indicating a partial mediation.

The total effect of power priming on implicit attitudes toward violence was significant, B = − 0.26, SE = 0.04, t(142) = − 7.24, *p* < .001. The indirect effect via state empathy was significant (indirect effect = − 0.10, 95% CI [− 0.15, − 0.05]). The significance of this indirect effect was confirmed as the 95% bias-corrected confidence interval did not contain zero, which is equivalent to a significance level of *p* < .05. Importantly, the direct effect of power priming on implicit attitudes remained significant after controlling for state empathy (direct effect = − 0.16, 95% CI [− 0.25, − 0.08]). These findings suggest that state empathy partially mediated the effect of power on implicit evaluations of violence. In other words, the activation of a sense of power was associated with less negative implicit attitudes toward violence, both directly and indirectly through reduced state empathy.

#### Manipulation check

An independent samples t-test confirmed the effectiveness of the power manipulation, with participants in the high-power condition reporting a significantly greater sense of power (*M* = 4.26 ± 1.17) than those in the low-power condition (*M* = 3.62 ± 1.14), t(144) = 3.36, *p* = .001, d = 0.55.

### Discussion

Study 1 showed that priming a sense of power was associated with differences in implicit evaluations of violence. Specifically, participants in the high-power condition exhibited significantly lower D-scores, indicating a weaker negative implicit evaluation of violence. This finding is consistent with previous research suggesting that experiences of power are associated with changes in automatic evaluative responses to violence [27].

Moreover, participants in the high-power condition reported significantly lower levels of state empathy, consistent with prior experimental work suggesting that power is often associated with reduced empathic responding [21, 24]. Mediation analysis indicated that state empathy partially mediated the relationship between power priming and implicit attitudes toward violence. Taken together, these findings are broadly consistent with theoretical perspectives such as the power paradox [46], suggesting that experiences of power may be associated with diminished interpersonal sensitivity. The present findings extend this perspective by indicating that even transient, situationally activated experiences of power may be accompanied by reduced momentary empathic responding.

## Experiment 2

Experiment 2 was designed to extend the findings of Experiment 1 by examining whether the effect of power on implicit evaluations of violence would replicate when power was activated through a conceptual priming task. In addition to testing the robustness of the power effect across different operationalizations, this study also examined whether individual differences in empathy shape the extent to which power priming influences implicit evaluations. Given that conceptual priming operates with minimal conscious awareness, its effects may be particularly likely to interact with individuals’ stable dispositional tendencies. Accordingly, this study focused on trait empathy as a dispositional moderator and tested whether individuals with higher levels of trait empathy would be less susceptible to the influence of power priming on implicit evaluations of violence.

### Method

#### Materials and instruments

##### Power priming

A word search task adapted from Yun and Li [47]was used to implicitly activate participants’ sense of power. Participants were presented with a 12 × 12 character grid and instructed to locate 18 embedded words. In the high-power condition, 13 of the target words were related to concepts of power (e.g., “control,” “authority,” “command”), whereas in the low-power condition, the key words reflected themes of powerlessness (e.g., “helpless,” “dependent”). The remaining 5 words were neutral in meaning (e.g., “green,” “clock”) and were included to balance the task.

##### Trait empathy

Trait empathy was assessed using the Empathic Concern subscale of the Interpersonal Reactivity Index [48]. This subscale consists of 7 items rated on a 7-point Likert scale, ranging from 1 (does not describe me well) to 7 (describes me very well). In the current study, the internal consistency of the scale was acceptable (α = 0.71).

##### Implicit attitudes toward violence

The same version of the Implicit Association Test (IAT) used in Study 1 was administered to assess participants’ implicit attitudes toward violence.

#### Procedure

Participants first completed the Empathic Concern subscale to assess trait empathy. They then performed the word search task, which served as the power priming manipulation. Finally, participants completed the Implicit Association Test (IAT) to measure their implicit attitudes toward violence. D-scores were computed using the same algorithm and procedures as in Study 1.

#### Participants

A total of 200 undergraduate students were recruited to participate in the study. After excluding 8 participants who failed to complete the tasks as instructed (e.g., provided irrelevant or extremely brief responses) and 14 whose error rates on the Implicit Association Test (IAT) exceeded 20% [44], the final sample consisted of 178 valid participants (101 females), with a mean age of 18.7 years (SD = 0.76). Participants were randomly assigned to either the high-power priming condition (*n* = 87, 47 females) or the low-power priming condition (*n* = 91, 54 females).

### Results

#### Moderating effect of trait empathy

We examined the moderating effect of trait empathy using PROCESS 4.0 for SPSS (Model 1, 5,000 bootstrap samples), controlling for age and gender. The analysis revealed a significant negative main effect of power priming on implicit evaluations of violence (B = − 0.70, SE = 0.21, t = − 3.42, *p* < .001, 95% CI [− 1.11, − 0.30]). The main effect of trait empathy showed a marginal effect (B = − 0.02, SE = 0.01, t = − 1.73, *p* = .085). Importantly, the interaction between power priming and trait empathy was statistically significant (B = − 0.02, SE = 0.01, t = 3.08, *p* = .002, 95% CI [0.01, 0.03]). This interaction accounted for an additional 4.6% of variance in IAT scores (ΔR² = 0.046), indicating that trait empathy moderated the effect of power priming on implicit evaluations of violence.

**Fig. 1:**
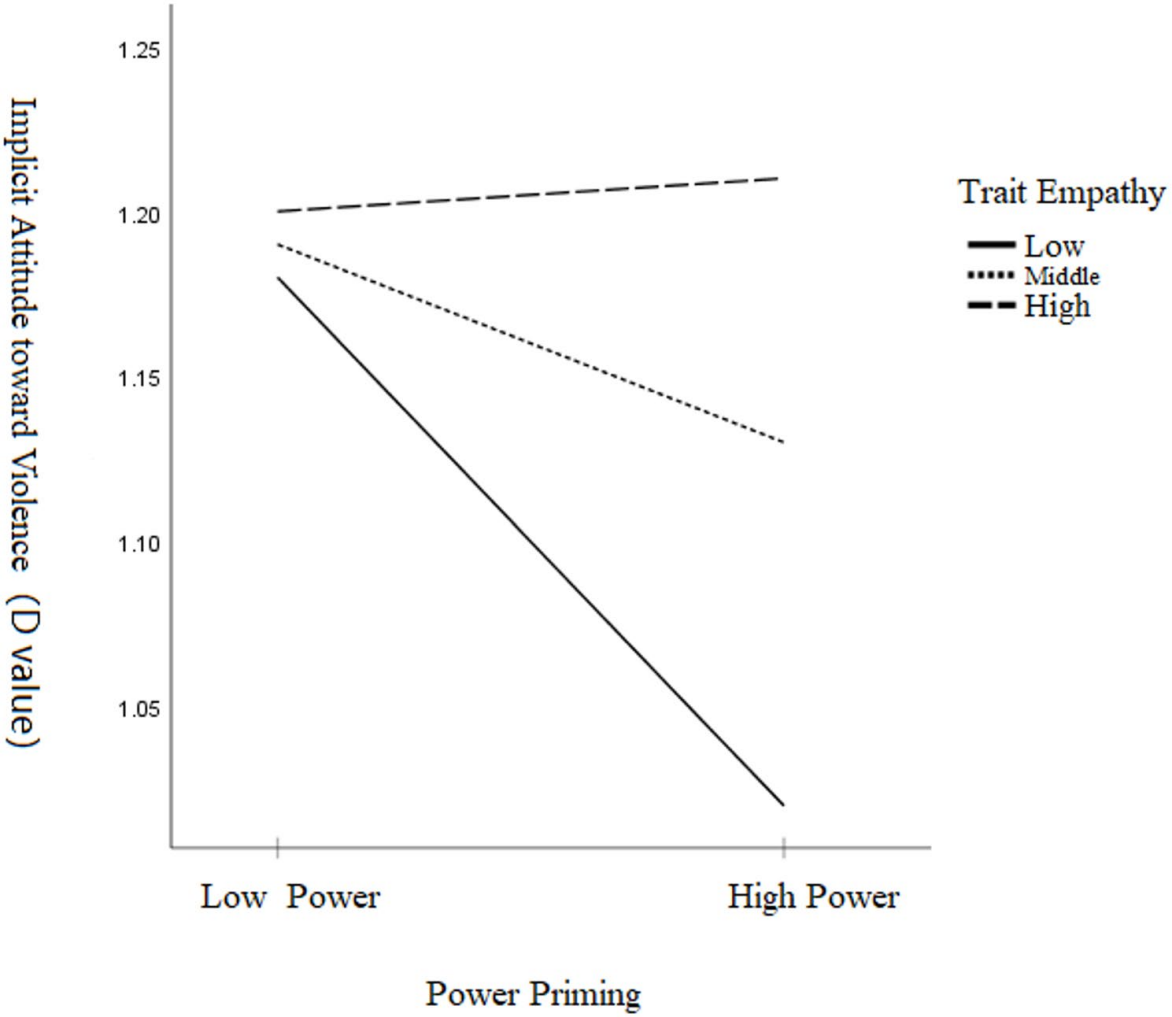
The Moderating Effect of Trait Empathy on the Relationship Between Power Priming and Implicit Attitudes Toward Violence

To further illustrate the moderating effect, a simple slopes analysis was conducted (Fig. 1). Based on trait empathy scores at the 16th percentile (score = 29), 50th percentile (score = 34), and 84th percentile (score = 38), participants were categorized as having low, moderate, and high levels of empathy, respectively [49]. The analysis revealed that power priming significantly reduced negative implicit evaluations of violence among participants with low trait empathy (B = − 0.16, *p* < .001). The effect remained significant, though weaker, at the moderate level of empathy (B = − 0.07, *p* = .039). In contrast, for participants with high trait empathy, the effect of power priming was non-significant (B = 0.01, *p* = .808).

**Fig. 2:**
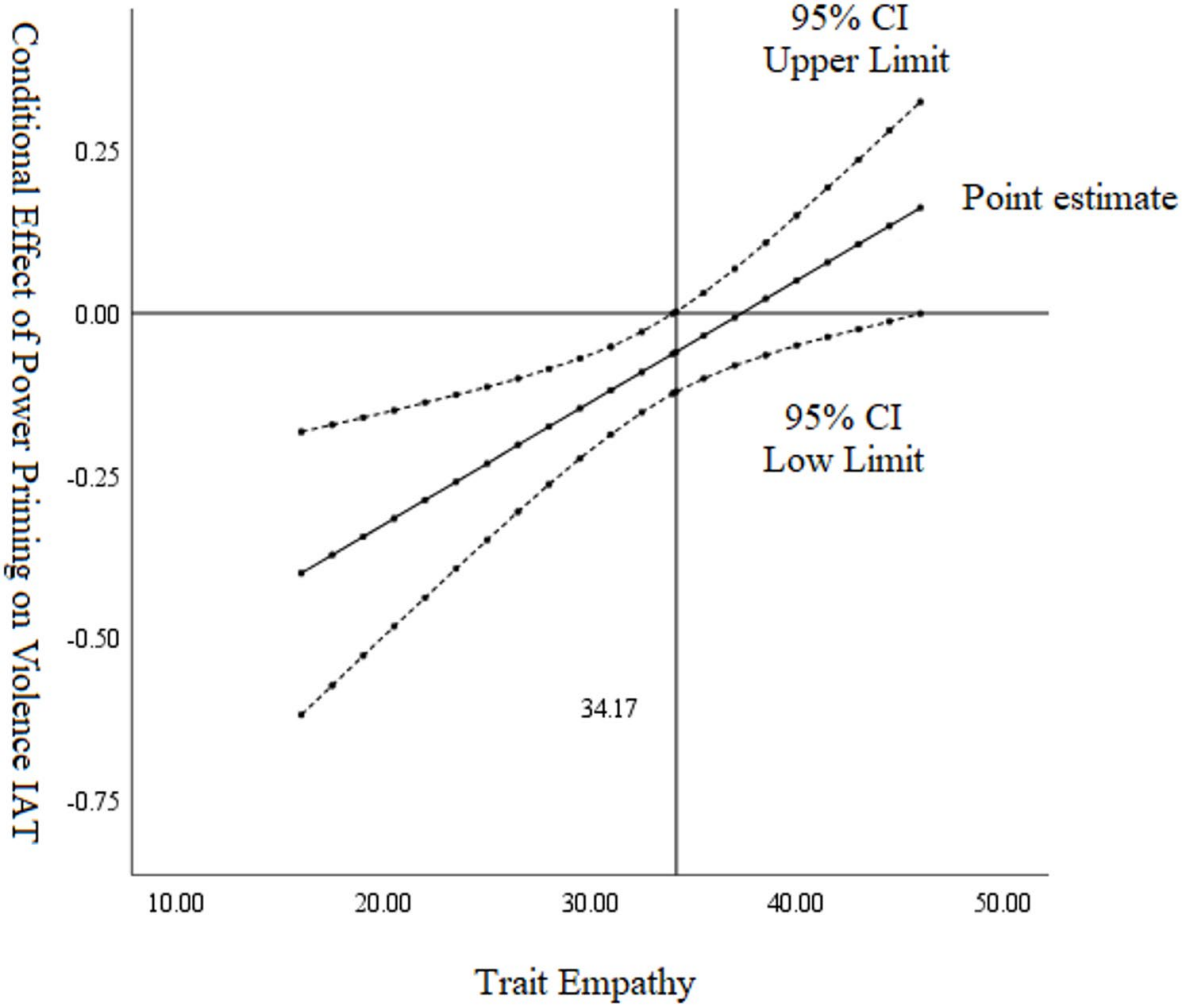
Johnson–Neyman plot of the conditional effect of power priming on implicit attitudes toward violence across levels of trait empathy. Note. The solid line represents the conditional effect (i.e., the regression coefficient) of power priming on violence IAT scores at different levels of trait empathy. The dashed lines represent the 95% confidence interval. Values of trait empathy below 34.17 correspond to regions where the conditional effect is statistically significant

To more precisely identify the range of trait empathy within which the moderating effect was significant, a Johnson–Neyman analysis was performed [50]. The results indicated that the conditional effect of power priming on implicit attitudes toward violence (i.e., the regression coefficient of power priming at different levels of trait empathy) was statistically significant when trait empathy scores were below 34.17, corresponding to approximately 56% of the sample. Beyond this threshold, the effect was no longer significant (Fig. 2). Inspection of the conditional effects and score distributions revealed no evidence of floor or ceiling effects in IAT scores across levels of trait empathy. This finding suggests that the influence of power priming on implicit evaluations of violence was limited to individuals with low to moderate levels of trait empathy, and did not extend to those with high levels of empathy.

#### Effect of power priming on implicit attitudes toward violence

A one-sample t-test against zero indicated that participants’ overall IAT D-scores were significantly greater than zero, t(177) = 68.99, *p* < .001, suggesting that the sample as a whole exhibited a reliable implicit negative association with violence. An independent samples t-test indicated that participants in the high-power priming condition showed significantly lower D-scores (M = 1.10, SD = 0.22) than those in the low-power condition (M = 1.19, SD = 0.22), t(176) = 2.71, *p* = .007, d = 0.41. This effect remained significant when age and gender were included as covariates in an ANCOVA, F(1, 174) = 6.82, *p* = .010, ηp² = 0.038. These results suggest that conceptual priming of power was associated with negative implicit evaluations of violence. This finding is consistent with previous research indicating an automatic cognitive association between power and violence [29, 42, 43].

### Discussion

Study 2 provided further evidence for the effect of power priming on implicit attitudes toward violence and highlighted the moderating role of trait empathy. While power priming was associated with lower negative implicit evaluations of violence, this effect was observed only among individuals with low to moderate levels of trait empathy. For individuals with high trait empathy, no significant differences emerged across power priming conditions.

Previous research suggests that heightened power is often associated with reduced empathy and moral sensitivity [21, 24]. However, the present findings extend this literature from a different angle, showing that trait empathy moderates individuals’ susceptibility to power priming. Specifically, individuals with low trait empathy—who are generally less attuned to others’ emotions—appeared more susceptible to the effects of power priming, showing a stronger tendency toward lower negative implicit evaluations of violence. In contrast, individuals with high trait empathy exhibited relative stability in their implicit evaluations across power conditions. In this sense, high trait empathy may function as a psychological buffer that mitigates impact of power on automatic evaluative responses toward violence.

## General discussion

This study examined how the sense of power and empathy influence individuals’ implicit attitudes toward violence. The findings revealed a complex interplay among power, empathy, and implicit cognitive evaluations of violence. With respect to the role of power, results indicated that nearly all participants demonstrated significant effects on the Violence IAT, reflecting a general tendency to show negative evaluative associations with violence. However, both the explicit sense of power (induced through autobiographical recall) and the implicit sense of power (activated through conceptual priming) significantly were systematically associated with differences in participants’ implicit attitudes. Specifically, individuals in the high-power conditions consistently exhibited lower D-scores compared to those in the low-power conditions. This pattern suggests that higher levels of experienced power are associated with weaker automatic activated negative evaluations of violence, as captured by the IAT. These findings are broadly consistent with the proposition that power is cognitively linked to violence [29, 42, 43], such that activating an individual’s sense of power may be accompanied by the activation of violent schemas or tendencies, which in turn may be associated with weaker negative implicit evaluations of violence.

The role of empathy in the relationship between power and implicit attitudes toward violence was examined across two studies, with a clear distinction drawn between state and trait empathy. Study 1 showed that explicit power priming was associated with lower levels of state empathy, which statistically mediated the association between power priming and implicit attitudes. Specifically, when individuals consciously experienced a heightened sense of power, their state empathy was lower, which was associated with less negative implicit evaluations of violence. In contrast, Study 2 revealed that trait empathy moderated the effect of power on implicit evaluations of violence: differences across power conditions were observed only among individuals with low to moderate levels of trait empathy. These findings suggest that, in real-world contexts where power may be implicitly activated, individuals’ dispositional empathy may play an important role in shaping their psychological responses.

This differentiation between state and trait empathy underscores the complex regulatory function of empathy in the power–violence relationship. Empathy can be transiently lower under conditions of situational power activation (state empathy), while trait empathy may function as a stable psychological buffer that determines an individual’s susceptibility to power’s influence. Regardless of form, empathy emerges as a central psychological variable connecting experiences of power with implicit violent attitudes.

It is also important to highlight the methodological distinction between the two studies in how power was manipulated. Study 1 utilized a situational recall task to elicit an explicit sense of power, which likely engaged more reflective, self-referential processing—making state empathy a salient mediator. In contrast, Study 2 employed conceptual priming to activate a more implicit sense of power, likely bypassing conscious emotional regulation and instead revealing the moderating influence of stable personality traits, such as trait empathy. More broadly, the adoption of a two-study design reflected a deliberate theoretical strategy: to establish a situational psychological mechanism in Study 1 and subsequently examine an individual-difference boundary condition in Study 2. This sequential approach is consistent with common practices in psychological research and helps clarify the distinct roles of state and trait empathy in the power–violence link. Methodologically, separating these constructs across independent studies also reduced the risk of participant fatigue, demand characteristics, and potential measurement contamination that might arise in overly complex single-study designs. Together, these complementary methodological approaches offer a more nuanced picture of how different forms of power activation relate to implicit evaluations of violence.

The primary contribution of this research lies in its integration of power, empathy, and implicit violent attitudes within a unified empirical framework. The findings suggest that the sense of power is systematically associated with implicit attitudes toward violence and interacts with empathy as both a mediating and moderating variable. This integrated model offers a novel psychological account of how power may shape implicit attitudes toward violence and highlights empathy as a promising target for intervention—particularly in contexts where power dynamics are salient.

Nonetheless, several limitations should be acknowledged. First, the present studies relied primarily on a single indirect outcome measure (the Violence IAT) and on specific experimental operationalizations of power (i.e., autobiographical recall and conceptual priming). Although these approaches are widely used and theoretically appropriate, future research would benefit from incorporating additional convergent measures (e.g., behavioral indices or alternative implicit tasks) and methodological triangulation to strengthen the robustness of the findings and to further disentangle power-specific effects from task-related influences. In addition, because no explicit attitude measure was included, the present conclusions are necessarily restricted to implicitly measured evaluations of violence rather than attitudes toward violence more broadly. Second, State empathy and trait empathy were examined in separate designs, offering a multi-level perspective on empathy’s role in the power–violence relationship. While this separation enhanced conceptual clarity and methodological rigor, future research could adopt integrative designs to examine how situational and dispositional empathy jointly shape responses to power. Third, the present findings were obtained within a specific cultural and situational context. Cultural meanings of power [51], as well as broader normative [52] or threat-related contexts [53], may shape how power is interpreted and enacted. Examining these contextual boundary conditions represents an important direction for future research.

## Conclusion

This study provides evidence that priming a sense of power is associated with reduced negative implicit evaluations of violence. The results suggest that this association operates through both mediating and moderating psychological processes. Specifically, activating an explicit sense of power lowers individuals’ state empathy, which in turn partially mediates the relationship between power and implicit evaluations of violence. Furthermore, trait empathy was found to moderate the effect of implicit power priming: the reduction in negative implicit evaluations of violence was observed only among individuals with low levels of trait empathy. These findings highlight the potential importance of empathy in shaping the psychological impact of power and suggest that both situational and dispositional empathy may regulate individuals’ implicit automatic evaluative responses to violence.

## Data Availability

The data that support the findings of this study are available from the corresponding author upon reasonable request.

## Abbreviations

ANCOVA: Analysis of Covariance
CI: Confidence Interval
SD: Standard Deviation
SE: Standard Error
SPSS: Statistical Package for Social Science
IAT: Implicit Association Test
